# Assessment of a continuous blood gas monitoring system in animals during circulatory stress

**DOI:** 10.1186/1471-2253-11-1

**Published:** 2011-01-11

**Authors:** Sandro Gelsomino, Roberto Lorusso, Ugolino Livi, Stefano Romagnoli, Salvatore Mario Romano, Rocco Carella, Fabiana Lucà, Giuseppe Billè, Francesco Matteucci, Attilio Renzulli, Gil Bolotin, Giuseppe De Cicco, Pierluigi Stefàno, Jos Maessen, Gian Franco Gensini

**Affiliations:** 1 Department of Heart and Vessels, Careggi Hospital, Florence, Italy; 2 Community Hospital, Brescia, Italy; 3 Santa Maria Della Misericordia Hospital, Udine, Italy; 4 University "Magna Graecia", Catanzaro, Italy; 5 Rambam Medical Center, Haifa, Israel; 6 Department of Cardiac Surgery, Academic Hospital, Maastricht, The Nederlands

## Abstract

**Background:**

The study was aimed to determine the measurement accuracy of The CDI™ blood parameter monitoring system 500 (Terumo Cardiovascular Systems Corporation, Ann Arbor MI) in the real-time continuous measurement of arterial blood gases under different cardiocirculatory stress conditions

**Methods:**

Inotropic stimulation (Dobutamine 2.5 and 5 μg/kg/min), vasoconstriction (Arginine-vasopressin 4, 8 and 16 IU/h), hemorrhage (-10%, -20%, -35%, and -50% of the theoretical volemia), and volume resuscitation were induced in ten swine (57.4 ± 10.7 Kg).Intermittent blood gas assessments were carried out using a routine gas analyzer at any experimental phase and compared with values obtained at the same time settings during continuous monitoring with CDI™ 500 system. The Bland-Altman analysis was employed.

**Results:**

Bias and precision for pO_2 _were - 0.06 kPa and 0.22 kPa, respectively (r^2 ^= 0.96); pCO_2 _- 0.02 kPa and 0.15 kPa, respectively; pH -0.001 and 0.01 units, respectively ( r^2 ^= 0.96). The analysis showed very good agreement for SO_2 _(bias 0.04,precision 0.33, r^2 ^= 0.95), Base excess (bias 0.04,precision 0.28, r^2 ^= 0.98), HCO_3 _(bias 0.05,precision 0.62, r^2 ^= 0.92),hemoglobin (bias 0.02,precision 0.23, r^2 ^= 0.96) and K^+ ^(bias 0.02, precision 0.27, r^2 ^= 0.93). The sensor was reliable throughout the experiment during hemodynamic variations.

**Conclusions:**

Continuous blood gas analysis with the CDI™ 500 system was reliable and it might represent a new useful tool to accurately and timely monitor gas exchange in critically ill patients. Nonetheless, our findings need to be confirmed by larger studies to prove its reliability in the clinical setting.

## Background

Blood gas monitoring is essential for the management of critically ill patients, providing valuable information about the state of the patient's oxygenation, gas exchange, ventilation and acid-base homeostasis [1].

Despite the rapidity of measurements and automation of modern blood gas analyzers (BGA), and the need for only small volumes of blood for any single sample, the intermittent nature of these measurements may provide only a snapshot of blood gases fluctuations occurring even in stable patients in the intensive care unit (ICU) [2]_. _This may result in potentially missing short-term trends, delaying adequate appraisal of ongoing metabolic, respiratory or cardiocirculatory changes, and, hence, limiting or impeding prompt therapeutic interventions. In addition the measurements may be inaccurate due to errors in sampling, storage and analysis [3].

Recent advances in technology have shifted the thrust from intermittent to continuous monitoring with the result that real time data are available continuously at the bedside [1].

The CDI™ Blood parameter monitoring system 500 (Terumo Cardiovascular Systems Corporation, Ann Arbor MI) is an optical fluorescence and reflectance-based in-line system which is used during cardiopulmonary bypass (CPB) to provide a reliable estimate of blood pCO_2_, pO_2_, pH and temperature with a 20s time-constant response [4]. However, whereas most of the published data on CDI™ 500 give evidence of the accuracy of this system during CPB [4,5], no information exist on its potential use as continuous blood gas monitoring at patient's bedside.

The aim of this study was to assess the accuracy and the reliability of the CDI™ 500 in the real-time continuous measurement of arterial blood gases under cardiocirculatory stress conditions in an animal model as compared to intermittent blood gas analysis.

## Methods

The study was approved by the Institutional Ethics Committee and animals were managed according to the principles of the "Guide for the Care and Use of Laboratory Animals" and according to the "Guide for the Care and Use of Laboratory Animals" and in accordance with the Italian national law (DL. 116/1992) and the recommendations of the European Community (86/609/CEE) for the care and use of laboratory animals.

Ten healthy swine, (mean weight Kg 57.4 ± 10.7), had preoperative intramuscular 15 mg/Kg ketamine (Parke Davis-Pfizer, Karlsruhe, DE) and 5 mg/Kg diazepam (Roche, Fontenay-sous Bois, France). General anesthesia was induced with intravenous ketamine (3.5 mg./Kg) and atropine sulfate 0.05 mg/Kg (Galenica Senese, Siena, IT). The trachea was intubated during spontaneous breathing and, after paralysis was obtained with 0.1 mg/Kg pancuromium bromide (N.V Organon, Oss, NL). The lungs were ventilated in a volume-controlled mode (Datex-Ohmeda; Helsinki; Finland) with 40% oxygen at 16-20 breaths per minute and a tidal volume of 8-10 ml/Kg adjusted to maintain partial carbon dioxide pressure ranging from 35 to 40 mmHg. Anesthesia was maintained with sevoflurane (2-3%).The electrocardiogram was continuously monitored in a standard D_II _lead and oxygen saturation was monitored by a continuous pulse oxymeter placed on the ear (Datex-Ohmeda; Helsinki; Finland).

An18-gauge cannula was inserted into the left carotid artery for intermittent arterial blood sampling, and blood gas analyses (ABL 825 Flex, Diamond Diagnostic, Holliston, MA) were carried out by FL: a total of 130 samples were analyzed. Any measurement was corrected by the animal's temperature.

**Figure 1:**
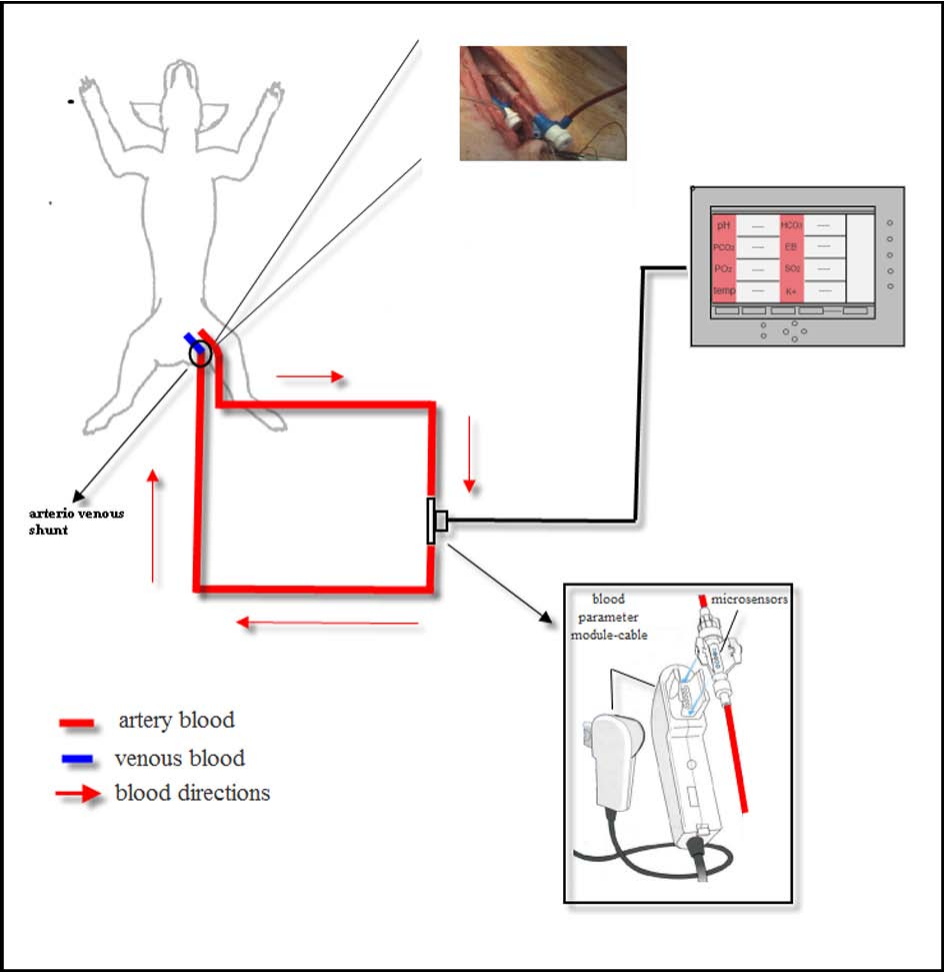
Schematic view of the dedicated CDI circuit (see text).

An 18-gauge and a 14-gauge cannula were inserted into the left femoral artery and the femoral vein, respectively, and an arterio-venous loop was created with a dedicated CDI™ 500 circuit (Figure 1) with a minimum blood flow of 35 ml/min into the heparin-treated shunt sensor CDI™ 510H.

The flow was precisely measured by a flow sensor incorporated into a ECLS pump drive (Rotaflow, Maquet, Rastatt, Germany) at different levels of arterial blood pressure (mean 60 ± 20 mmHg). Body temperature, measured with a rectal probe, was maintained between 37.5° and 38°C by means of a heating blanket and by warming the infused solutions. The system updates measurements every six seconds. A single investigator (FL) collected and stored in a computer system (Acer Aspire Travel Mate 1690; Acer-Italia, Milan, Italy) data from blood samples and the corresponding values displayed on the CDI system at any time the blood was taken.

The right femoral artery was cannulated with a standard 18-gauge catheter and connected with the Most-Care™ monitor powered by Pressure Recording Analytical Method (PRAM)[6] via a standard pressure monitoring set (Edwards Lifesciences, Irvine, CA, USA). The resulting signal was processed by Most-Care™ (Release 1.00A,Vytech Healthcare, Padova, Italy) for the determination of CO. Most-Care™ was connected to a corresponding laptop computer (Acer Aspire Travel Mate 1690; Acer-Italia, Milan, Italy) for continuous recording of the hemodynamic data (Most-Care Smart Card reader, Vytech Health™, Padova, Italy).

The PRAM has been validated either in experimental [6] or clinic studies in unstable patients [7] and it resulted to be reliable under different hemodynamic conditions.

The right internal jugular vein was cannulated with a 14-gauge multi-lumen catheter (Three Lumen Central Venous Catheterization Set. Arrow International, Inc.2400 Bernville Road, Reading, PA 19605 USA) and the distal lumen connected to a standard pressure transducer.

### Experimental Protocol

Thirty minutes after the end of preparation, the experimental protocol started and three different hemodynamic challenges were induced in the following temporal order:

1) Inotropism, tachycardia and vasodilation (mimicking a physically-related stress test - dobutamine infusion).

2) Peripheral vascular vasoconstriction (Arginine-vasopressin infusion).

3) Hemorrhagic shock (controlled blood loss).

4) Volume cardiocirculatory resuscitation (controlled fluid loading).

Before starting the dobutamine phase, a baseline blood sample was taken (B_1_). The dobutamine phase was performed in two separate 30 minute steps: 2.5 mg/kg/min (D_1_) and 5 mg/kg/min (D_2_). Dobutamine infusion was then stopped and, when the heart rate returned within 10% of that measured in B_1_, a new blood sample was taken during this re-established stable condition (B_2_) which was maintained for one hour. Then, three 30 minute steps of vasoconstriction were induced with arginine-vaso-pressin (AVP) at the doses of 4 IU/h (AVP_1_),8 IU/h (AVP_2_), and 16 IU/h (AVP_3_). After one hour from the end of arginine-vasopressin infusion and normalization of the mean arterial pressure a new blood gas analysis was carried out (B_3_). Hemorrhage was obtained with a controlled bleeding obtaining four progressive steps of exsanguinations: H_1_: -10% circulating blood,H2 : -20%, H_3 _: -35%, and H_4 _: -50% of the theoretical volemia Blood volume was estimated as: animal weight (Kg) × 65 ml/Kg. The blood volume was withdrawn at a rate that decreased over time in a stepwise fashion with half of the target hemorrhage volume occurring in the first 5 min and the remaining volume over the next 10 min[8]. At 20 min, resuscitated pigs were administered hydroxyethyl starch 6% (volume resuscitation, VR). Additional infusions of 5 ml/kg were provided at 30, 60, 120, and 180 min if hypotension (MAP < 60 mmHg) or tachycardia (HR > baseline value)were observed.

### The Device

The CDI™ blood parameter monitoring system 500 consists of a monitor to process and display data, a user-selected combination of blood parameter modules (BPM_s_), and hematocrit/oxygen saturation probe (H/S probe), disposable sterile sensor, H/S cuvette and a calibrator.

The disposable sensors and/or H/S cuvette are installed in the cable heads of the BPM_s _or the H/S probe, at a point of the circuit will allow adequate exposure to blood. BPM_s _,which measures Ph, pCO_2, _pO_2 _and K^+^, use optical fluorescence technology in conjunction with the disposable CDI™ system 500 shunt sensor. The H/S probe, which measures hematocrit, hemoglobin and oxygen saturation, uses optical reflectance technology in conjunction with the disposable H/S cuvette.

Sensors for Ph, pCO_2, _pO_2 _are calibrated using the CDI calibrator 540 and two canisters of calibration gases which contain precise, defined levels of pCO_2, _and pO_2 _[9]. To perform the calibration the system measures the intensities emitted by a micro sensor as it is exposed to Gas A and Gas B. It them plots these two fluorescent measurements as a function of the predefined values of the calibration gases. The system uses the two points to create a slope and a y-intercept for that parameter. The measures the fluorescent intensity of the blood and it uses the slopes and intercept to extrapolate corresponding blood parameter values.

Calibration of K^+ ^also relies on a two-point slope and intercept calibration process. The slope is defined using the factory-measured value encoded in the calibration code entered from the sensor pouch during the initial calibration sequence. The intercept point is obtained using the K^+ ^level in an animal blood sample processed using a laboratory analyzer. The value is then entered in the CDI™ 500 system to replace the stored reading. Finally, the H/S probe is pre-calibrated at the factory for oxygen saturation, hematocrit and hemoglobin. The system does not require further re-calibration[9].

### Statistical Analysis

All the data were analyzed with Stats Direct (Relase.2.5.8, Cheshire, UK) and Graph Pad Prism (Release4.0; San Diego, USA). Hemodynamic variables at each time point were tested for significant differences by ANOVA for repeated measures and Bonferroni's correction was also applied for a *post-hoc *analysis. Agreement between intermittent and continuous blood gas analyses was assessed with a Bland-Altman plot [10] obtaining: bias (mean difference between methods), precision (± 2 SD of bias) and limits of agreement. The relationship between the techniques was investigated by linear regression analysis and Pearson's correlation coefficient.

## Results

**Figure 2:**
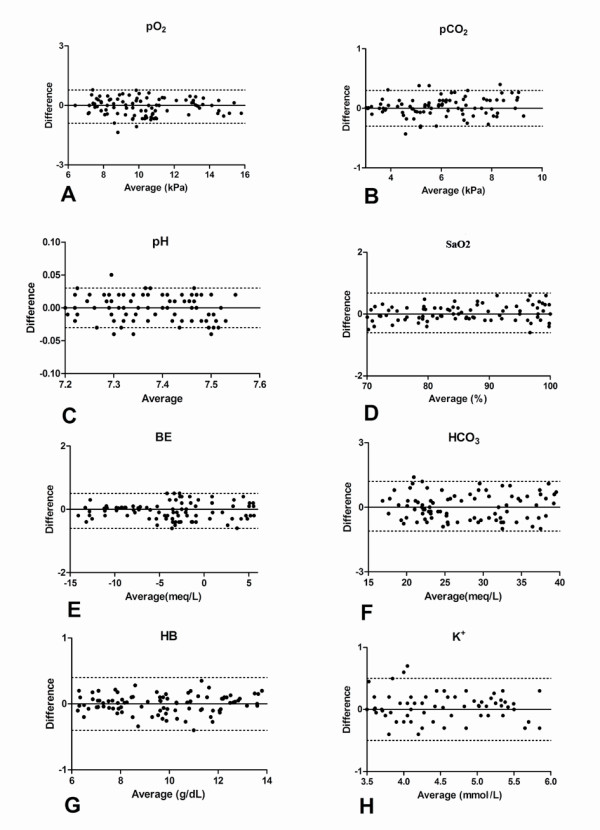
**Bland Altman plots showing all measurements**. Data are plotted as differences versus means for **(A) **pO_2 _(Partial Pressure of Oxygen). **(B) **pCO_2 _(Partial Pressure of Carbon-dioxide). **(C) **pH.**(D) **SaO_2 _(Oxygen saturation)**. (E) **BE **(**Base Eccess). **(F) **HCO_3 _(Bicarbonate).**(H) **K^+^(Potassium ione) **(G) **Hb (Hemoglobin).The solid lines represent the Bias the dashed lines the precision (± 2 SD)

Hemodynamic changes during the experiment are shown in Table 1. Table 2 displays results of Pearson's correlation and Bland-Altman analysis: for pO_2_, the linear regression between the two methods was r^2 ^= 0.96. Bias and precision were - 0.06 kPa and 0.22 kPa, respectively. The analysis of different hemodynamic phases showed a significant correlation at concurrent point of the experiment. The Bland-Altman analysis is depicted in Figure 2A. The graphical analysis shows an overall homogeneity between the two methods even at lower arterial pO_2 _levels. pCO_2 _measurement revealed a linear regression of r^2 ^= 0.95 between CDI™ and blood-gas analyzer. Bias and precision were - 0.02 kPa and 0.15 kPa, respectively. The Bland-Altman plot (Figure 2B) showed optimal agreement at corresponding pCO_2 _values during hemodynamic variations.

**Table 1:** Hemodynamic Parameters

	Overall	**B**_**1**_	**D**_**1**_	**D**_**2**_	**B**_**2**_	**AVP**_**1**_	**AVP**_**2**_	AVP_**3**_	**B**_**3**_	**H**_**1**_	**H**_**2**_	**H**_**3**_	**H**_**4**_	VR
**CO**_**T**_**(L/min)**	3.8 ± 1.08	4.3 ± 1.18	4.93 ± 1.5	4.77 ± 1.9	4.17 ± 1.06	3.92 ± 0.96	3.50 ± 1.16	3.17 ± 0.98 *	4.2 ± 0.82	3.8 ± 0.73	2.63 ± 0.67	2.38 ± 0.74	1.9 ± 0.55*	5.17 ± 1.85^†‡§ ^^||^
**SV (ml)**	44.3 ± 11.52	56.9 ± 14.94	48.8 ± 20.33	40.1 ± 17.95 *	52.1 ± 12.92	54.6 ± 12.71	51.9 ± 11.61	50.4 ± 8.78	59.8 ± 12	45.2 ± 9.2*	29.8 ± 7.9^#^	26.1 ± 7.9^#†^	17 ± 4.1^#**‡‡^	60.2 ± 9.5^††‡§#^
**HR (bts/min)**	85.7 ± 17.9	77.8 ± 23.3	104.8 ± 19.2 *	124.6 ± 14.2 ^†^	81.1 ± 16	72.5 ± 14.1	67.3 ± 16.9 ^‡^	62.7 ± 16^†^	69.1 ± 15.6	84 ± 21.2	88 ± 21.1^§^	91.1 ± 18.8*	105.8 ± 16.8^#|||| ‡‡^	85.1 ± 20.2 ^##^
**MAP (mmHg)**	55.1 ± 7.03	55.5 ± 3.48	65.1 ± 12.4	60.3 ± 11.9	52 ± 3.5	65.6 ± 8.7*	73.8 ± 9^†^	76.1 ± 6.7^† §^	64 ± 8.7	45.1 ± 4.7^#^	36.8 ± 6.3^# †^	33 ± 4.4^# †^	29.6 ± 5^# ||||^	59.1 ± 6.6^||||‡ §||^
**CVP (mmHg)**	8.82 ± 1.46	9.83 ± 1.99	9.83 ± 1.60	9.50 ± 1.22	9.67 ± 2.42	11.50 ± 1.38	11.67 ± 0.82	11.67 ± 0.82	9.6 ± 1.8	8.8 ± 1.7	6.3 ± 1.6	4.6 ± 1.8*^†^	1.1 ± 0.7^# ^**	10.6 ± 1.2^‡‡§||^
**SVR (dyne/s/cm**^**5**^**)**	1123 ± 346	912 ± 263	947 ± 323	913 ± 288	857 ± 249	1195 ± 475	1532 ± 460^†^	1755 ± 522^† §^	1199 ± 246	971 ± 382	1040 ± 377	1081 ± 352	1323 ± 301	870 ± 258 ^##^

**Abbreviations**: B: Basal; D: Dobutamine; CO: Cardiac Output; SV: Stroke Volume; HR: Heart Rate; MAP: Mean Arterial Pressure; CVP: Central Venous Pressure; SVR: Systemic Vascular Resistances. AVP: Arginine-vasopressin.

Significance at repeated measures analysis of variance. *p < 0.05 vs. B_1-3_. ^†^p < 0.001 vs. B_1-3_. Significance at repeated measures analysis of variance. *p < 0.01 vs. B_4_. ^†^p < 0.001 vs. B_4_. ^‡^p < 0.05 vs. B_4_. ^§^p < 0.05 vs. AVP_1_. Significance at repeated measures analysis of variance. *p < 0.01 vs. B_5_. ^†^p < 0.05 vs. H_1_. ^‡^p < 0.001 vs. H_2_. ^§^p < 0.001 vs. H_3_. ^||^p < 0.001 vs. H_4_. ^#^p < 0.001 vs. B_5_. **p < 0.001 vs. H_1_. ^††^p < 0.01 vs. H_1_. ^‡‡^p < 0.05 vs. H_2_. ^§§^p < 0.05 vs. B_5_. ^||||^p < 0.01 vs. H_1_. ^##^p < 0.05 vs. H_4_

**Table 2:** Pearson Correlation Coefficient and Bland-Altman analysis

	All	**B**_**1-3**_	**D**_**1-2**_	**AVP**_**1-3**_	H1	H2	H3	H4	VR
**Ph**									
**r**^2^*	0.9604	0.9216	0.9025	0.8649	0.9776	0.8441	0.9781	0.9409	0.9604
**Bias**	-0.001	-0.01	0.002	-0.01	0.004	0.01	-0.001	-0.008	-0.003
**Precision**	0.01	0.009	0.023	0.02	0.02	0.01	0.008	0.009	0.01
**LoA**	-0.03 to 0.03	0.02 to 0.01	-0.04 to 0.04	-0.04 to 0.03	-0.04 to 0.03	-0.01 to 0.04	-0.01 to 0.01	-0.02 to 0.01	-0.02 to 0.01
**BE**									
**r**^2^*	0.9801	0.9216	0.9744	0.9929	0.9925	0.9801	0.9801	0.9444	0.9801
**Bias**	-0.04	0.08	0.05	-0.01	-0.01	-0.03	-0.02	-0.07	0.02
**Precision**	0.28	0.33	0.31	0.10	0.12	0.26	0.32	0.29	0.3
**LoA**	-0.61 to 0.58	-0.57 to 0.74	-0.6 to 0.6	-0.24 to 0.27	-0.35 to 0.33	-0.30 to 0.33	-0.36 to 0.51	-0.45 to 0.40	-0.5 to 0.5
**pO**_**2**_									
**r**^2^*	0.9604	0.9409	0.9025	0.9216	0.9409	0.8724	0.8134	0.8649	0.9604
**Bias**	-0.06	-0.09	-0.09	-0.11	-0.02	-0.10	-0.04	-0.10	0.01
**Precision**	0.22	0.13	0.16	0.16	0.24	0.31	0.23	0.27	0.11
**LoA**	-0.28 to 0.36	-0.30 to 0.37	-0.26 to 0.29	-0.42 to 0.58	-0.4 to 0.30	-0.23 to 0.26	-0.45 to 0.42	-0.18 to 0.27	-0.42 to 0.49
**pCO**_**2**_									
**r**^2^*	0.9560	0.9246	0.7241	0.9036	0.9909	0.9409	0.9281	0.9744	0.9801
**Bias**	0.02	-0.05	-0.12	0.04	-0.01	0.06	0.02	0.08	0.07
**Precision**	0.15	0.27	0.54	0.09	0.09	0.06	0.15	0.18	0.19
**LoA**	-0.24 to 0.33	-0.21 to 0.15	-0.61to 0.49	-0.13 to 0.22	-0.04 to 0.14	-0.15 to 0.18	-0.25 to 0.30	-0.12 to 0.20	-0.14 to 0.22
**SaO**_**2**_									
**r**^2^*	0.9564	0.9725	0.9744	0.9241	0.9755	0.9925	0.9056	0.9929	0.9464
**Bias**	0.04	-0.02	-0.01	-0.88	-0.02	0.01	0.04	0.01	0.04
**Precision**	0.33	0.34	0.33	0.41	0.20	0.30	0.34	0.26	0.2
**LoA**	-0.6 to 0.7	-0,2 to 0.5	-0.1 to 0.3	-0.3 to 0.6	-0.08 to 0.3	-0.07 to 0.2	-0.6 to 0.6	-0.08 to 0.23	-0.3 to 0.3
**HCO**_**3**_									
**r**^2^*	0.9201	0.8724	0.8976	0.9025	0.9241	0.9604	0.9329	0.9604	0.9801
**Bias**	0.05	0.09	0.08	0.06	0.07	-0.18	0.07	0.18	0.11
**Precision**	0.62	0.69	0.51	0.70	0.62	0.64	0.59	0.42	0.45
**LoA**	-1.1 to 1.2	-1.2 to 1.4	-1.1 to 1.0	-1.2 to 0.7	-1.2 to 1.5	-1.4 to 1.0	-1.3 to 1.5	-0.6 to 1.0	-0.7 to 1.1
**Hb**									
**r**^2^*	0.9601	0.9849	0.9801	0.9836	0.9804	0.9649	0.9216	0.9649	0.9801
**Bias**	0.02	0.02	0.03	0.03	0.01	0.02	0.05	0.01	0.01
**Precision**	0.23	0.30	0.22	0.30	0.13	0.27	0.42	0.18	0.2
**LoA**	-0.2 to 0.2	-0.2 to 0.3	-0.1 to 0.2	-0.5 to 0.6	-0.1 to 0.1	-0.1 to 0.1	-0.6 to 0.6	-0.2 to 0.2	-0.1 to 0.1
**K+**									
**r**^2^*	0.9364	0.9941	0.9869	0.9569	0.9329	0.8836	0.7281	0.7056	0.8464
**Bias**	0.02	0.01	-0.02	0.03	-0.04	-0.05	0.06	0.10	0.05
**Precision**	0.27	0.12	0.34	0.29	0.32	0.33	0.49	0.59	0.34
**LoA**	-0.3 to 0.3	-0.2 to 0.2	0.4 to 0.4	-0.3 to 0.3	-0.3 to 0.4	-0.5 to 0.5	-0.5 to 0.5	-0.8 to 0.8	-0.4 to 0.4

**Abbreviations**: B: Basal; DBT: Dobutamine; AVP: Arginine-vasopressin; H: Hemorrhage; VR: Volume Resuscitation;; BE: Base Eccess ( meq/L); pO_2_: Partial Pressure of Oxygen (KPascal); pCO_2_: Partial Pressure of Carbon-dioxide (KPascal); SaO_2_: Oxygen saturation; (%) HCO_3_: Bicarbonate (meq/L); Hb: Hemoglobin (g/dL); K^+:^: Potassium ione (mmole/L).

All recorded CDI™ sensor-related values for pH have been plotted versus the corresponding laboratory blood gas analyzer according to Bland-Altman analysis (Figure 2 C) and showed a very minimal bias and precision of -0.001 and 0.01pH units, respectively (r^2 ^= 0.96).

The analysis of the different hemodynamic phases including all measurements showed very good agreement for SO_2_(Figure 1D, bias 0.04,precision 0.33, r^2 ^= 0.95), Base excess (Figure 1E, bias 0.04,precision 0.28, r^2 ^= 0.98), HCO_3 _(Figure 1F, bias 0.05,precision 0.62, r^2 ^= 0.92),hemoglobin (Figure 1G, bias 0.02,precision 0.23, r^2 ^= 0.96) and K^+ ^(Figure 1H, bias 0.02,precision 0.27, r^2 ^= 0.93). The sub-analysis of K^+ ^during hemorrhage showed a good agreement in H_1 _and H_2 _while in H_3 _and H_4 _although the agreement was lower, the correlation was still significant (p = 0.02).

## Discussion

Management of critically ill patients requires frequent arterial blood gas analyses in the assessment of respiratory and hemodynamic function [2,11].The current standard for blood gas analysis is intermittent blood gas sampling with measurements performed in vitro, in a blood gas analyzer [12]. Nonetheless, intermittent arterial blood gas analysis provides only snapshots of information about the patient's status, may involve a significant turnaround time, exposes the health-care professionals to the patient's blood and results in iatrogenic blood loss [1,13,14].

Several attempts have been made to overcome the disadvantages of intermittent arterial blood sampling and to develop a real-time continuous blood gas monitoring system.

Furthermore, pulse oximetry, capnometry and transcutaneous blood gas measurement cannot fully replace arterial pO_2_, arterial pCO_2 _and arterial pH analyses in the clinical setting because of their significant limitations[15-20].

Advances in optical sensor technology have allowed the development of devices that can monitor blood gas levels using optical sensors (optodes) [21]. The extra-arterial blood gas (EABG) monitors such as the CDI™ 2000 (3M Healthcare, Tustin, CA) are on-demand systems which utilize optodes which are externally attached to the arterial catheter. Although studies have demonstrated their accuracy and precision compared with conventional blood gas analyzers [22,23], the on-demand monitoring systems are not continuous and the rate of measurements is operator-dependent.

Unlike EABG systems, intra-arterial blood gas (IABG) monitors have the sensors placed into the arterial blood stream. Contrasting results have been reported about the reliability of their clinical performance[24-32] and their consistency and reliability are argued because of the brittleness of the fibers [33] and their drawbacks related to the intra-arterial environment and the indwelling sensor (wall effect, kinking and bending impact of wrist position) [1]. Although significant improvement in the performance of the new IABG monitors have been reported [34,35], concerns still remain about the accurateness of pO_2 _analysis especially during hemodynamic instability [33].

The CDI™ 500 (Terumo Cardiovascular Systems Corporation, Ann Arbor MI) is a microprocessor-based monitor which uses an optical fluorescence technology to measure blood gases, pH and potassium. In addition it employs an optical reflectance technology to measure oxygen saturation, hematocrit and hemoglobin. This system is widely employed for continuous blood gas monitoring during cardiopulmonary bypass [4,5,36,37]. As far as we are aware, it has never been employed for continuous bedside monitoring of blood gases.

Therefore we tested the "off label" use of this device in a preclinical model in different hemodynamic conditions for a future investigation in the clinical practice. The Clinical Laboratory Improvement Amendments for in vitro arterial blood gas analyzers (CLIA) of 1988 [38] indicates limits of precision as follows: pH: ± 0.04; pCO2: ± 0.66 (4.9) kPa (mmHg); pO2: ± 8%. In this study, the sensor showed excellent performance, with values lower than the CLIA limits. Furthermore, even though results are difficult to compare because of different study design, different sites of sensor placement (radial artery, femoral artery or brachial artery) and dissimilar length of measurements, our figures compare favorably with data reported in the literature [22,34,39-41].

It has been shown that pO_2 _is the most flow-dependent variable [34] and IABG monitors may not always be accurate in determination of arterial blood oxygenation especially during hemodynamic instability [33]. Indeed, the improvement in the accuracy of pO_2 _measurements is an important focus for the development of such equipment. In our experience, the CDI™ 500, whatever the test conditions, showed a good agreement with the intermittent gas analysis. We showed similar results for Base Excess (precision,0.28), SO2 (precision,0.33), HCO3 (precision,0.62), Hemoglobin (precision,0.23) and potassium (precision,0.27). Finally, the precision was maintained throughout the study and under disparate cardiocirculatory stress conditions

The CDI™ 500 was easy to set up and to calibrate and it did not need re-calibration. In contrast, the available systems for continuous blood gas monitoring require re-calibration (using in vitro laboratory gas determinations) after prolonged monitoring in the clinical setting according to the recommendations of the manufacturer. However, the issue of re-calibration was not discussed in previous studies, and it was performed at different time points [34].

Furthermore, because of the heparin-treated sensor, there was no need for a continuous heparinised saline flush, necessary in intra-vascular systems to maintain the patency of the circuit [1].The flush solution, at room temperature, contains dissolved oxygen and carbon dioxide at partial pressures similar to that of the atmosphere thus it may alter the local gas tension. As a consequence, the system may measure the blood gas variables in the flush solution, resulting in errors known as the "flush effect" [1,42]. The CDI™ 500 was highly reliable even when the flow decreased significantly during vasoconstriction in contrast with IABG systems which are susceptible to blood flow and when it is low or stops measurements become unreliable [1].

### Perspective of Clinical Application

In our opinion CDI™ 500 might be extremely useful in the clinical setting, particularly in patients with low cardiac output, requiring a close monitoring. In these cases, CDI 510H sensoring device would be advisably inserted in a femoral artery-to-vein configuration to maintain a 35 ml/min flow through the circuit. In patients with preserved cardiac output it is possible to design a radial artery-to-any vein configuration provided that the vein catheter is at least a 16-gauge (internal jugular catheter) to guarantee an optimal intra-device flow.

Finally, it must be taken into consideration that such a methodology might be applied for continuous and on-line monitoring particularly in severely critical patients, like the ones on cardiocirculatory support (Extracorporeal Membrane Oxygenation or Ventricular Assist Devices). In these circumstances, CDI circuit can be instrumented either along the arterial or the vein ECMO circuit line to continuously measure blood parameter enabling a real-time appraisal of ongoing efficacy of peripheral perfusion and, thus, a prompt warning and call for reassessment in case of suboptimal parameters without waiting the next external blood gas analysis.

Nonetheless further studies will be required to make this device into a viable clinical approach. In particular the following issues need to be addressed:

a) The true advantages of this system over intermittent or near-continuous monitoring in terms of blood loss, need for erythrocyte transfusions (especially in infant and children)and the risk of nosocomial infections.

b) The hazards of arterio-venous shunting of blood especially of clinical importance in critically ill patients with low cardiac output.

c) The accuracy of the device and need for re-calibration after use for long times.

d) Need of heparinization should be further investigated before a devices' routine clinical use, though, in the present experience, we had no clotting-related problems

### Limitations

Our study presents some limitations that should be pointed out:

First, optode devices intrinsically have lower signal to noise ratio at higher PO2; thus the

nice agreement shown here for a lower range of PO2 may not hold up as well in

a high range. For this reason our findings are not fully comparable with prior studies

that did investigate a full range of PaO2.

Second, cost effectiveness is an important issue when new monitoring devices are introduced in the clinical practice. No cost-benefit analysis was carried out in this study and is subject of ongoing research.

## Conclusions

On the basis of our experience we can conclude that continuous blood gas analysis with the CDI™ 500 system was practical, accurate and reproducible. It might represent a new useful tool to monitor gas exchange in critically ill patients, even on mechanical circulatory support, and during anesthesia for major surgery. Nonetheless our findings need to be confirmed by larger studies to prove its reliability in the clinical setting.

## Key Messages

• Intermittent gas analysis may provide only a snapshot of blood gases fluctuations occurring even in stable patients in the intensive care unit.

• The extra-arterial blood gas monitors are not continuous and the rate of measurements is operator-dependent.

• The intra-arterial blood gas monitors present some drawbacks related to the intra-arterial environment and the indwelling sensor.

• The CDI™ Blood parameter monitoring system 500 is an optical fluorescence and reflectance-based in-line system which is used during cardiopulmonary bypass

• The "off label" use of The CDI™ 500 in a preclinical model was reliable. Thus it might represent a new useful tool to accurately and timely monitor gas exchange in critically ill patients.

## Abbreviations

BGA: blood gas analyzers; ICU: intensive care unit; AVP: arginine-vaso-pressin; VR: volume resuscitation; BPM_s_: blood parameter modules; H/S hematocrit/oxygen saturation probe (probe); EABG: extra-arterial blood gas; IABG: intra-arterial blood gas; CLIA: Clinical Laboratory Improvement Amendments for in vitro arterial blood gas analyzers; CO: Cardiac Output; SV: Stroke Volume; HR: Heart Rate; MAP: Mean Arterial Pressure; CVP: Central Venous Pressure; SVR: Systemic Vascular Resistances. BE: Base Eccess; pO_2_: Partial Pressure of Oxygen; pCO_2_: Partial Pressure of Carbon-dioxide; SaO_2_: Oxygen saturation;HCO_3_: Bicarbonate; Hb: Hemoglobin; K^+^: Potassium ione.

## Competing interests

The authors declare that they have no competing interests.

## Authors' contributions

SG and RL participated in the study design acquisition of the data, drafted the manuscript. FL SR, GDC, PLS, FM and RC managed the acquisition of data. UL, AR and GB participated in the design of the study.

GFG and JM participated in the design of and revised the draft for critical content.

All authors read and approved the final manuscript.

## Pre-publication history

The pre-publication history for this paper can be accessed here:

http://www.biomedcentral.com/1471-2253/11/1/prepub
